# The QuinteT Recruitment Intervention supported five randomized trials to recruit to target: a mixed-methods evaluation

**DOI:** 10.1016/j.jclinepi.2018.10.004

**Published:** 2019-02

**Authors:** Leila Rooshenas, Lauren J. Scott, Jane M. Blazeby, Chris A. Rogers, Kate M. Tilling, Samantha Husbands, Carmel Conefrey, Nicola Mills, Robert C. Stein, Chris Metcalfe, Andrew J. Carr, David J. Beard, Tim Davis, Sangeetha Paramasivan, Marcus Jepson, Kerry Avery, Daisy Elliott, Caroline Wilson, Jenny L. Donovan, By-Band-Sleeve study group, Chris A. Rogers, Robert Andrews, Jane M. Blazeby, James Byrne, Jenny L. Donovan, Jamie Kelly, Graziella Mazza, David Mahon, Hamish Noble, Barnaby C. Reeves, Janice L. Thompson, Sarah Wordsworth, Richard Welbourn, CSAW study group, David Beard, Andrew Carr, Jonathan Cook, Cushla Cooper, Benjamin Dean, Jenny L. Donovan, Alastair Gray, Stephen Gwilym, Andrew Judge, Naomi Merritt, Jane Moser, Jonathan Rees, Ines Rombach, Julian Savulescu, Irene Tracey, Karolina Wartolowska, HAND-1 study group, Eleanor Harrison, Wei Tan, Nicola Mills, Alexia Karantana, Kirsty Sprange, Lelia Duley, Daisy Elliott, Jane M. Blazeby, William Hollingworth, Alan A. Montgomery, Tim Davis, Optima prelim study group, Rob Stein, John Bartlett, David Cameron, Amy Campbell, Peter Canney, Jenny L. Donovan, Janet Dunn, Helena Earl, Mary Falzon, Adele Francis, Peter Hall, Victoria Harmer, Helen Higgins, Louise Hiller, Luke Hughes-Davies, Claire Hulme, Iain Macpherson, Andreas Makris, Andrea Marshall, Christopher McCabe, Adrienne Morgan, Sarah Pinder, Christopher Poole, Elena Provenzano, Daniel Rea, Nigel Stallard, the Romio feasibility study group, Kerry N.L. Avery, C. Paul Barham, Richard Berrisford, Jane M. Blazeby, Jenny L. Donovan, Jackie Elliott, Stephen J. Falk, Rob Goldin, George Hanna, Andrew A. Hollowood, Richard Krysztopik, Chris Metcalfe, Sian Noble, Grant Sanders, Christopher G. Streets, Dan R. Titcomb, Tim Wheatley

**Affiliations:** a Population Health Sciences, University of Bristol, Canynge Hall, 39 Whatley Road, Bristol BS8 2PS, Bristol, United Kingdom; b NIHR Collaboration for Leadership in Applied Health Research and Care at University Hospitals Bristol NHS Foundation Trust, Bristol, United Kingdom; c Clinical Trials and Evaluation Unit, Bristol Royal Infirmary, School of Clinical Sciences, University of Bristol, Bristol, United Kingdom; d University College London Hospitals (UCLH), Biomedical Research Centre (BMC), University College London Hospitals, 1st Floor Central, 250 Euston Road, London NW1 2PG, UK; e Nuffield Department of Orthopaedics, Rheumatology and Musculoskeletal Sciences, University of Oxford, Oxford, United Kingdom; f Royal College of Surgeons Surgical Intervention Trials Unit (SITU), University of Oxford, Oxford, United Kingdom; g Queen's Medical Centre, Nottingham University Hospitals NHS Trust, Derby Road, Nottingham NG7 2UH, UK

**Keywords:** Randomized controlled trial, Clinical trial, Recruitment, Training healthcare professionals, Qualitative research, Communication

## Abstract

**Objective:**

To evaluate the impact of the QuinteT Recruitment Intervention (QRI) on recruitment in challenging randomized controlled trials (RCTs) that have applied the intervention. The QRI aims to understand recruitment difficulties and then implements “QRI actions” to address these as recruitment proceeds.

**Study Design and Setting:**

A mixed-methods study, comprising (1) before-and-after comparisons of recruitment rates and the numbers of patients approached and (2) qualitative case studies, including documentary analysis and interviews with RCT investigators.

**Results:**

Five UK-based publicly funded RCTs were included in the evaluation. All recruited to target. Randomized controlled trial 2 and RCT 5 both received up-front prerecruitment training before the intervention was applied. Randomized controlled trial 2 did not encounter recruitment issues and recruited above target from its outset. Recruitment difficulties, particularly communication issues, were identified and addressed through QRI actions in RCTs 1, 3, 4, and 5. Randomization rates significantly improved after QRI action in RCTs 1, 3, and 4. Quintet Recruitment Intervention actions addressed issues with approaching eligible patients in RCTs 3 and 5, which both saw significant increases in the number of patients approached. Trial investigators reported that the QRI had unearthed issues they had been unaware of and reportedly changed their practices after QRI action.

**Conclusion:**

There is promising evidence to suggest that the QRI can support recruitment to difficult RCTs. This needs to be substantiated with future controlled evaluations.

**What is new?**

**Key findings**
• The QuinteT Recruitment Intervention (QRI) supported five challenging randomized controlled trials (RCTs) in surgery/oncology to recruit to target. The QRI operated by first investigating the sources of recruitment difficulty in each RCT and then implementing tailored “QRI actions” to address the issues as recruitment proceeded.
• Four of the five RCTs encountered recruitment challenges, underpinned by difficulties in communicating the trial to potential participants and issues around approaching eligible patients. These issues were addressed through QRI actions delivered as the RCTs were recruiting.
• Randomization rates significantly improved after QRI action in three trials that encountered communication difficulties. There was no significant difference in two RCTs, which both recruited above target from their outsets and had received previous QRI support. Two trials that received QRI actions designed to address issues around approaching patients saw significant improvements in the numbers of patients approached per site/month.

**What this adds to what was known?**
• There is a growing body of empirical research devoted to explaining why recruitment can be challenging, but a dearth of effective solutions. Numerous systematic reviews have repeatedly highlighted the paucity of broadly applicable, effective strategies to improve trial recruitment. This initial evaluation suggests that the QRI can effectively identify and address recruitment issues, leading to improvements in recruitment in a range of RCTs.

**What is the implication and what should change now?**
• This evidence provides a platform for trialists to consider adopting the QRI (or similar approaches) in their RCTs, particularly if recruitment issues are anticipated. Future controlled evaluations of the QRI are needed to build upon this promising evidence.

## 1 Introduction

High-quality evidence about treatment effectiveness is crucial for improving patient outcomes and promoting judicial use of resources. Well-conducted randomized controlled trials (RCTs) can provide this evidence, but one of the biggest threats to an RCT's success is suboptimal recruitment. This can compromise a trial's statistical power, delay implementation of findings, and lead to costly extensions or early closure [1], [2]. Various guidance and tools (e.g., PRECIS-2) are now available to support trial design and planning [3], [4], but only half of RCTs meet their recruitment targets [5], and recruitment issues remain the top reason for premature closure [1], [6]. The threat of recruitment difficulties can also discourage important RCTs from being attempted.

There is a growing body of empirical research devoted to understanding RCT recruitment issues, but a paucity of effective solutions [7], [8], [9], [10]. The little work that has been conducted has predominantly focused on lessons learned from previous RCTs [10]. Promising initiatives, such as the MRC “Start” program and the clinical trials transformation initiative, aim to empirically test interventions designed to support recruitment [11], [12], but interventions evaluated to date have had a narrow field of application or emerged as ineffective [8]. There remains an urgent need for widely applicable interventions to facilitate RCT recruitment [7], [8], particularly for challenging RCTs, such as those comparing very different approaches to managing life-changing conditions.

The QuinteT (qualitative research integrated into trials) Recruitment Intervention (QRI) was designed to optimize RCT recruitment [13]. It evolved over the course of 2 decades since its inception in the ProtecT study—a challenging RCT that randomized men to surgery, radiotherapy, or monitoring for localized prostate cancer [14], [15]. Rather than relying on fixed strategies, the QRI delivers tailored solutions to improve recruitment, based on a detailed understanding of trial-specific challenges. The intervention focuses on how trial information is communicated to potential participants and seeks to identify and address other factors that may undermine recruitment (e.g., difficulties approaching potential participants). The QRI has been implemented in a growing number of challenging RCTs [16], [17], [18], [19], [20], some of which have now completed and reported [21], [22], [23].

The aim of this study was to evaluate the impact of the QRI on RCT recruitment. We undertook a mixed-methods evaluation of all main and pilot RCTs that have integrated the intervention and completed (or stopped) recruitment to date. Our primary objective was to investigate the impact of the QRI on recruitment rates. Secondary objectives were to explore the QRI's likely mechanisms of action and identify opportunities for its refinement.

## 2 Materials and methods

### 2.1 Overview of the QRI

The QRI is designed to be integrated with an RCT's recruitment plans. It aims to rapidly understand recruitment issues and then implement solutions to address these while recruitment is underway. This is achieved through two iterative phases [13]. In brief:

• Phase 1 is a rapid investigation of the RCT's recruitment processes using four core data collection processes: (1) audio-recording appointments where recruiters discuss the trial with potential participants; (2) interviews with recruiters and trial management group (TMG) members; (3) mapping eligibility and recruitment pathways; and (4) scrutinizing trial documentation. The findings from each source are triangulated to determine the key areas of recruitment difficulty and fed back to the chief investigator and TMG.
• Phase 2 involves designing and implementing a “plan of action” to address recruitment difficulties. The plan comprises one or more “QRI actions,” typically including confidential feedback for recruiters who audio-record their appointments, group feedback/training, written “Tips and Guidance,” and changes to trial documentation and processes (e.g., patient information leaflets, recruitment pathways). QuinteT Recruitment Intervention actions are designed and delivered collaboratively by the QRI researcher(s) and the TMG.

### 2.2 Study design and sampling

A mixed-methods evaluation of the QRI was undertaken, comprising a quantitative before-and-after comparison of recruitment rates and a qualitative case study analysis of each RCT's recruitment process.

Randomized controlled trials eligible for inclusion needed to have integrated the full QRI and be closed to recruitment (or have completed pilot/feasibility stages). All RCTs that had collaborated with the QuinteT team between 2005 and 2017 were assessed, and fulfillment (or not) of each QRI component was recorded by L.R. and cross-checked by researchers who worked on each RCT (see Supplementary Information 1).

### 2.3 Data collection and analysis

#### 2.3.1 Documentary analysis

The documentary analysis was conducted first, to produce an overview of the recruitment issues identified through the QRI and the QRI actions implemented to address these. Each eligible RCT's protocol, funding application, and articles/reports were consulted for information about the trial and QRI (see Supplementary Information 2).

#### 2.3.2 Quantitative analysis

Recruitment logs maintained by clinical trials units (CTUs) recorded the number of eligible patients approached per month, and the number of those who went on to be randomized, for each site within each RCT. Site-specific data were not available for RCT 4, so total monthly recruitment figures were used instead. Data from only one of the two sites in RCT 2 were included because recruitment log information was not available for the other site. All RCTs recorded the number of patients randomized per month, apart from RCT 3, which referred to the “number of patients consented”; we therefore used “consent” as a surrogate for randomization for this trial.

“QuinteT Recruitment Intervention actions” were defined as any initiatives to optimize recruitment that had been informed by phase 1 QRI findings, including study-wide actions (e.g., dissemination of written guidance on how to explain the trial) or site-specific activities (e.g., feedback to a recruiter). As the timing and nature of the first QRI action could differ across sites in a given RCT, patients approached in the time up to and including the month of the first “QRI action” (in their trial and site) were considered part of the “preintervention” period; patients approached in any month after were part of the “postintervention” period. In the absence of site-specific data for RCT 4, we conservatively assumed that all QRI actions impacted all sites.

The analysis was conducted by an independent statistician (L.J.S.) blind to the RCTs' identities. The analysis plan was determined before accessing the data. All statistical analyses were performed in Stata 15.1.

Analysis 1 investigated randomization success (whether a patient who was eligible and approached went on to be randomized). The association between preintervention/postintervention and randomization success was analyzed using a mixed-effects logistic regression model, with preintervention/postintervention and trial fitted as fixed effects, and site as a random effect. Results are presented as odds ratios (ORs) with associated confidence intervals (CIs). Whether the effect of preintervention/postintervention differed by trial was investigated by fitting a “preintervention/postintervention by trial” interaction term in the model; if the interaction was significant at the *P* = 0.05 level, then results were presented by trial rather than overall.

Analysis 2 examined the number of patients approached per site in the preintervention and postintervention periods. The association between preintervention/postintervention and the number of patients approached per site/month was analyzed using a mixed Poisson regression model, with preintervention/postintervention and trial fitted as fixed effects, adjusting for the number of months of recruitment at a site as the exposure, and site fitted as a random effect. Results were presented as incident rate ratios (IRRs) with associated CIs. Again, results were presented by trial if the preintervention/postintervention by trial interaction was significant at the *P* = 0.05 level.

#### 2.3.3 Qualitative analysis

Semistructured interviews were conducted to investigate how the QRI had been implemented in each RCT as a means of triangulating the documentary analysis findings. Interviews also explored trialists' perceptions of the strengths and limitations of the QRI. A key informant sampling approach was used, where RCT TMG members who had been involved in recruitment were identified and invited (via email) to participate. Interviews were conducted between November 2016 and August 2017 via telephone or in person and audio-recorded with permission. A topic guide was used to consistently cover topics aligned with the objectives (Supplementary Information 3). Written consent had already been obtained through the QRIs, and interviewers (L.R. and S.H.) verbally confirmed individuals were happy to participate.

Interviews were transcribed verbatim and analyzed through constant comparative approaches adopted from Grounded Theory [24], [25]. This was an iterative process, involving line-by-line coding of data and arrangement of codes into themes and subthemes as new data were obtained and previously coded transcripts were revisited. NVivo (V10, QSR International) was used to organize the data. Interviews were analyzed by L.R., with a subset double-coded by a researcher who had not worked on any of the trials (C.C.). “Negative” cases that appeared to contradict emerging themes were sought and presented in the findings where apparent.

## 3 Results

Thirty RCTs were considered for inclusion, with start dates spanning 2005-2017. Fifteen RCTs were excluded because they had not finished recruitment at the time of the evaluation, and 10 were ineligible because they did not implement the full QRI (see Supplementary Information 4 for reasons why).

**Fig. 1:**
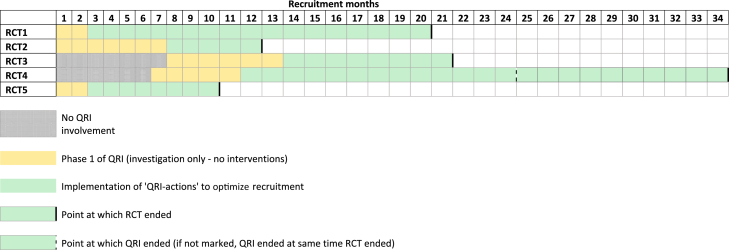
Timeline of RCTs, with points of QRI integration and point at which QRI actions were implemented. RCT, Randomized controlled trial; QRI, QuinteT Recruitment Intervention.

Five RCTs were included in the evaluation. All were pragmatic, publicly funded trials in UK secondary care settings, spanning different clinical specialties and comparing very different treatments, including less/no treatment (Table 1). Four had integrated the QRI from the outset due to anticipated recruitment challenges, and one RCT integrated the QRI partway through, in response to recruitment difficulties. Figure 1 shows the timeline for each RCT, including when the QRI started (initiation of phase 1) and when QRI actions started to be implemented (phase 2). The timing of the first QRI action ranged from month 3 to month 14 of the RCT's recruitment period.

**Table 1:** Details of the RCTs included in the evaluation

RCT identifier	Feasibility or main study	Clinical specialty	Trials arms	Number of sites	Duration of recruitment period (mo)	Point of QRI integration
RCT 1 (ISRCTN00786323)	Feasibility (internal pilot)	Bariatric surgery	Surgical procedure A vs Surgical procedure B	2	20	Outset
RCT 2 (ISRCTN59036820)	Feasibility (external pilot)	Surgery for esophageal cancer	Surgical procedure A vs Surgical procedure B	2^a^	12	Outset
RCT 3 (ISRCTN42400492)	Feasibility (external pilot)	Oncology (breast cancer)	Drug treatment vs “Test-directed” drug treatment	35	21	Incorporated into the RCT protocol from the outset but began on month 8 of recruitment period.
RCT 4 (ISRCTN 33864128	Main	Orthopedic surgery (shoulders)	Assessment/monitoring vs surgery vs placebo surgery	32	34	Incorporated into the RCT protocol partway through the recruitment period; began in month 8.
RCT 5 (ISRCTN11164292)	Feasibility	Orthopedic surgery (hands)	Surgical procedure A vs Surgical procedure B	3	10	Outset

*Abbreviations*: RCT, Randomized controlled trial; QRI, QuinteT Recruitment Intervention.

a Although the RCT took place across two sites, only one of these provided screening log data, and thus only one site was included in this evaluation.

Two trials (RCT 2 and RCT 5) had received support from QuinteT before recruitment started. Randomized controlled trial 5 received up-front training/guidance, including a generic presentation on recruitment challenges/solutions at the trial's launch, customized “tips and guidance” documents for explaining the RCT, refinements to patient-facing literature, and some recruiters had attended a generic QuinteT recruitment workshop [26]. Randomized controlled trial 2 recruiters had been exposed to QRI feedback in a previous trial [27]. This prior support did not fulfill the definition of “QRI actions” because activities were not informed by trial-specific investigation of recruitment issues. Therefore, the periods up to the first “QRI action” were still considered “preintervention” in these trials.

### 3.1 Recruitment outcomes

All RCTs successfully recruited their target samples. Four achieved this on time or earlier than planned, without opening additional sites (RCTs 1, 2, 3, and 5). Randomized controlled trial 4, which integrated the QRI partway through its funded period, had an extension and opened eight additional sites.

#### 3.1.1 Analysis 1: changes in randomization success

**Fig. 2:**
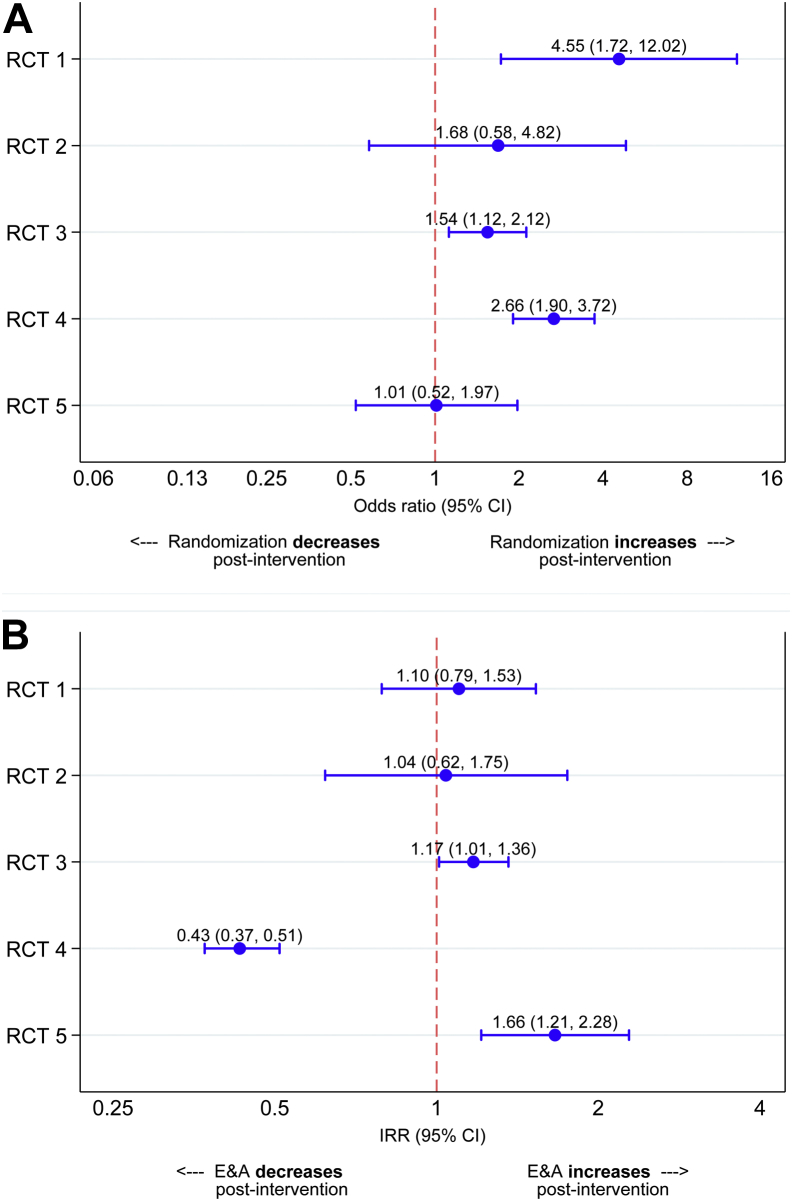
Results of analysis 1 (change in randomization success, preintervention and postintervention) and analysis 2 (changes in the numbers of patients approached, preintervention and postintervention). Panel A: Odds ratio of randomization success after the first QRI action, shown for individual trials. Panel B: Incident rate ratios (IRR) of eligible and approached (E&A) patients after the first QRI action, shown for individual trials. RCT, Randomized controlled trial; QRI, QuinteT Recruitment Intervention.

Based on pooled data across the RCTs, 307/783 (39%) of approached eligible patients were randomized before the first QRI action, compared with 582/1,160 (50.2%) after (Table 2). The effect of preintervention/postintervention differed for the individual RCTs (preintervention/postintervention by trial interaction *P* = 0.018), and so each trial was considered individually in the model (Figure 2, panel 1). Randomization success significantly improved after intervention for RCT 1 (OR 4.55, 95% CI 1.72-12.02), RCT 3 (OR 1.54, 95% CI 1.12-2.12), and RCT 4 (OR 2.66 (95% CI 1.90-3.72). There was no evidence of a difference in RCT 2 (OR 1.68, 95% CI 0.58-4.82) and RCT 5 (OR 1.01, 95% CI 0.52-1.97).

**Table 2:** Numbers and proportions of eligible and approached patients randomized in the preintervention period (before the first QRI action) and postintervention period, shown for all trials combined and each individual trial

	Number of E&A patients (*N*)	Number of randomized patients (n)	Proportion of E&A patients randomized (n/*N*)
All RCTs combined			
Preintervention period	783	307	39.2%
Postintervention period	1,160	582	50.2%
Period following:			
1-2 QRI actions	518	246	47.5%
3-4 QRI actions	237	117	49.4%
5+ QRI actions	405	219	54.1%
RCT 1			
Preintervention period	40	5	12.5%
Postintervention period	304	119	39.1%
Period following:			
1-2 QRI actions	120	47	39.2%
3-4 QRI actions	61	24	39.3%
5+ QRI actions	123	48	39.0%
RCT 2			
Preintervention period	55	22	40.0%
Postintervention period	19	10	52.6%
Period following:			
1-2 QRI actions	13	8	61.5%
3-4 QRI actions	6	2	33.3%
5+ QRI actions	-	-	-
RCT 3			
Preintervention period	382	164	42.9%
Postintervention period	363	186	51.2%
Period following:			
1-2 QRI actions	260	127	48.8%
3-4 QRI actions	103	59	57.3%
5+ QRI actions	-	-	-
RCT 4			
Preintervention period	230	80	34.8%
Postintervention period	396	232	58.6%
Period following:			
1-2 QRI actions	49	29	59.2%
3-4 QRI actions	65	32	49.2%
5+ QRI actions	282	171	60.6%
RCT 5			
Preintervention period	76	36	47.4%
Postintervention period	78	35	44.9%
Period following:			
1-2 QRI actions	76	35	46.1%
3-4 QRI actions	2	0	0%
5+ QRI actions	-	-	-

*Abbreviations:* E&A, eligible and approached; RCT, Randomized controlled trial; QRI, QuinteT Recruitment Intervention.

#### 3.1.2 Analysis 2: changes in the number of patients approached per site/month

The pooled data across all RCTs showed that 1.7 patients were approached per site/month in the preintervention periods, compared with 1.3 patients per site/month in the postintervention periods (Table 3). Considering the trials separately (preintervention/postintervention by trial interaction *P* < 0.001; Figure 2, panel 2), the number of patients approached significantly decreased after intervention in RCT 4 (IRR 0.43, 95% CI 0.37-0.51), did not change in RCT 1 (IRR 1.10, 95% CI 0.79-1.53) and RCT 2 (IRR 1.04, 95% CI 0.62-1.75), and showed a significant improvement in RCT 3 (IRR 1.17, 95% CI 1.01-1.36) and RCT 5 (IRR 1.66, 95% CI 1.21-2.28).

**Table 3:** Average number of patients eligible and approached per site per month in the preintervention and postintervention period for each RCT and for all RCTs combined

	Number of site mo	Number of E&A patients	Average number of E&A patients (per site per mo)
All RCTs combined			
Preintervention period	453	783	1.7
Postintervention period	900	1,160	1.3
RCT 1			
Preintervention period	4	40	10
Postintervention period	33	304	9.2
RCT 2^a^			
Preintervention period	9	55	6.1
Postintervention period	3	19	6.3
RCT 3			
Preintervention period	265	382	1.4
Postintervention period	227	363	1.6
RCT 4			
Preintervention period	157	230	1.5
Postintervention period	626	396	0.6
RCT 5			
Preintervention period	18	76	4.2
Postintervention period	11	78	7.1

*Abbreviations:* RCT, Randomized controlled trial; E&A, eligible and approached.

a Site data available from one of the 2 recruiting sites.

### 3.2 Recruitment obstacles

Table 4 lists the challenges identified in each RCT. Recruitment issues fell under two categories:

**Table 4:** Summary of key sources of recruitment difficulty based on phase 1 findings and timing/type of QRI actions to address difficulties

RCT identifier	Key sources of recruitment difficulties to emerge from phase 1 investigation	Timing (recruitment mo) and type of phase 2 QRI actions
RCT 1	1) Difficulties integrating the RCT into existing clinical service: a Complex recruitment pathways (from point of identifying eligible patients to the recruitment appointment) b Structure of consultations was often not conducive to explaining the RCT rationale; RCT participation was not presented as a viable option. 2) Difficulties conveying equipoise when describing trial arms to patients 3) Tendency to accept patient preferences, without exploration 4) Difficulties articulating and explaining trial concepts (e.g., “randomization”)	• Month 3: individual feedback for site 1 • Month 4: group feedback for site 1 • Month 7: individual feedback for site 1 • Month 8: group feedback for site 1 • Month 9: individual feedback for site 2 • Month 11: individual feedback for site 1 • Month 13: individual feedback for site 2 • Month 14: individual feedback for site 2 • Month 16: “Tips and guidance” document circulated to all sites • Month 18: individual feedback for site 1 • Month 18: Research nurse training event (both sites)
RCT 2	Recruitment was perceived to be progressing well throughout, and recruiters were encouraged to continue their practices. Additional tips were provided in relation to: 1) Conveying equipoise during recruitment appointments (when describing trial arms) 2) Explaining trial concepts (e.g., “randomization” and “blinding”) 3) Addressing patient preferences	• Month 8: Group feedback for site 2 • Month 9: Individual feedback for 2 recruiters in site 1 • Month 11: Group feedback for site 1 (clinicians) • Month 11: “Tips and guidance” document circulated to all sites
RCT 3	1) Clinicians' discomfort approaching the full spectrum of eligible patients (clinicians' individual sense of equipoise). 2) Clinicians' and research nurses' discomfort around introducing the study, based on assumptions that patients would not be able to process the offer of trial participation. 3) Structure of consultations was not conducive to explaining the RCT rationale; RCT participation was not presented as a viable option. 4) Difficulties articulating uncertainty and conveying equipoise during recruitment appointments 5) Difficulties explaining the trial design clearly 6) Tendency to accept patient preferences, without exploration 7) Difficulties articulating and explaining trial concepts (e.g., “randomization” and “blinding”)	• Month 14: “Tips and guidance” document circulated to all sites • Month 15: Edited patient information leaflet circulated to all sites • Month 15: Group feedback presented to region 1 (attended by five sites). • Month 17: Group feedback presented to region 2 sites, with discussion of hypothetical vignettes (attended by seven sites) • Month 18: Individual feedback provided for recruiter from one site • Month 19: Individual feedback provided for recruiters from 3 sites • Month 19: Group feedback to two sites.
RCT 4	1) Difficulties articulating and explaining the trial treatments/management approaches 2) Difficulties conveying equipoise during recruitment appointments (when describing trial arms) 3) Difficulties explaining the trial design 4) Tendency to accept patient preferences, without exploration	• Month 13: Individual feedback • Month 14: Individual feedback • Month 15: “Tips and guidance” in study-wide newsletter • Month 16: Study-wide group feedback, attended by 15 sites • Month 16: Site visit to provide recruitment training (one site) • Month 17: Site visits to provide recruitment training (two sites) • Month 22: Site visits to provide recruitment training (two sites) • Month 22: “Tips and guidance” document circulated to all sites
RCT 5	1) Complex recruitment pathways (from point of identifying eligible patients to the recruitment appointment) 2) Discomfort approaching the full spectrum of eligible patients (equipoise difficulties). 3) Difficulties conveying equipoise during recruitment appointments (when describing trial arms) 4) Tendency to accept patient preferences, without exploration	• Month 6: Site-specific group feedback delivered to sites 1 and 2 • Month 6: Individual feedback provided to recruiters in sites 1 and 2. • Month 7: Adapted “tips and guidance” document sent to site 3. • Month 9: Individual feedback to recruiter in site 2. • Month 10: Visit to site 2 to provide training for new recruiters

#### 3.2.1 Issues relating to communicating the trial to eligible patients

Recruitment to most trials (RCTs 1, 3, 4, and 5) was undermined through difficulties in explaining the RCT to potential participants (“communication issues”). These included equipoise issues, apparent through recruiters' difficulties in articulating uncertainty, and imbalanced descriptions of the trial arms (where particular treatments were conveyed as being superior or more appropriate). Difficulties addressing patient preferences for particular treatments/approaches were also common, but most problematic in RCT 1, where the majority (>87%) of patients approached declined the trial in favor of a preferred treatment. Audio-recorded appointments revealed a tendency for recruiters to elicit patients' preferences at the start of the consultation and then accept these without discussing the RCT.

More specific issues arose in individual trials, such as difficulties in articulating trial designs. For example, RCT 3 randomized patients to routine treatment versus “test-directed” treatment, whereby patients received a test that was hypothesized to inform their optimal treatment (i.e., “personalized treatment”). Patients sometimes found it difficult to comprehend the distinction between randomization and “testing,” both of which played a role in determining final treatment prescribed. Design-related issues also arose in RCT 4, where surgical recruiters encountered difficulties in articulating placebo and nonsurgical arms.

As an exception, RCT 2 revealed few communication issues to address in the preintervention stage; QRI actions therefore encouraged recruiters to continue their practices, with some suggestions for additional information provision geared toward better informing patients.

#### 3.2.2 Issues pertaining to the number of patients approached

Recruitment pathways varied from one site to another and were inefficient in some sites, as in RCT 5. Interviews with recruiters from RCT 5 and RCT 3 also indicated that some recruiters felt uncomfortable offering the trial to eligible patients who they felt would be more appropriate for one treatment over another; this appeared to restrict the numbers of eligible patients approached.

### 3.3 QRI actions to optimize recruitment

The QRI actions implemented in each RCT are shown in Table 4. Feedback on recruiters' communication practices was at the heart of each trial's “plan of action” to optimize recruitment, delivered to individuals or groups of recruiters (e.g., a single center or multiple centers). Feedback used anonymized extracts from recruitment appointments/interviews to illuminate difficulties and potential solutions, focusing on issues around equipoise communication, articulation of RCT designs, and management of patient preferences. Group feedback sessions offered opportunities for peer-to-peer discussion; individual feedback offered more specific advice, bespoke to a recruiter's practices.

Study-wide QRI actions to address communication issues were delivered in all RCTs through trial-specific “tips and guidance” documents. These included recommendations for structuring consultations and explaining common concepts (e.g., “randomization”). Changes to patient-facing documentation were also implemented in some RCTs with a view to conveying equipoise and trial design more clearly (e.g., RCT 3).

Issues around approaching patients were addressed by reviewing summaries of screening logs and exploring cases where eligible patients had not been approached. In RCT 3, hypothetical vignettes were used to prompt discussion around contentious aspects of the eligibility criteria to encourage peer-to-peer discussion. The intention here was to enable recruiters who were less comfortable approaching patients with certain characteristics to gain reassurance from peers who were more comfortable [17]. Scrutiny of patient pathways and suggestions for refining how patients were identified/approached were also discussed in site-specific feedback sessions in RCT 5.

### 3.4 Overall impact of QRI actions

Randomization success improved in RCTs 1, 3, and 4, all of which appeared to be compromised by communication issues. Communication issues were also apparent in RCT 5, but there was no difference in randomization success (although the trial recruited above target throughout its duration). There was also no significant improvement in randomization success in RCT 2, although the documentary analysis indicated that QRI actions encouraged recruiters to maintain their current successful practices. Randomized controlled trial 2 and RCT 5 recruited above target throughout and both had been exposed to prior QuinteT training.

The numbers of patients approached per site/month improved after intervention in RCTs 3 and 5, in line with documented strategies designed to address issues around approaching patients. No significant improvements were observed in the other RCTs, but there were no QRI actions that specifically targeted approach issues. The number of eligible patients approached per site/month significantly decreased in RCT 4. Informal correspondence with the TMG indicated that fewer eligible patients were being identified in the later stages of this trial, reportedly due to depletion of prevalent cases. The qualitative interviews also indicated that the new centers opened during the postintervention period did not engage as well as original centers, which diluted the number of patients approached per site/month.

### 3.5 Trialists' perspectives on the QRI

Seven trial investigators representing all five RCTs participated in interviews. Their accounts of the QRI activities generally complemented the documentary data. The one exception to this was RCT 2. Although the documentation indicated that the mandatory components of the QRI had been conducted, interviews suggested that the QRI was not fully implemented as it was not deemed necessary (following observation of successful recruitment trends). Randomized controlled trial 2 did not have a dedicated QRI researcher, and the QRI activities were not conducted intensively. This was corroborated by researchers who contributed to RCT 2's QRI, both of whom questioned whether RCT 2 was eligible for the evaluation. Informants' accounts from other trials indicated that the QRI had been delivered as reported in the documentary sources.

Trial investigators were forthright about the difficulties of quantifying the impact of the QRI but most felt that QRI actions translated into improved recruitment (Web Appendix 1). Most discussed how the QRI had introduced them to sources of difficulty that they had previously been unaware of, and those with direct experience of recruiting gave specific examples of how the QRI had changed their discussions with patients. Individual/group feedback and written guidance were thought to have catalyzed these changes:

Investigator 4: In the very early days, I was a very poor recruiter. […]. …. And actually I learnt from [the QRI], and the work [QRI researcher] did. Because toward the end I was much more successful, and I had really started to follow the guidance in the tips document. And I think it was extremely useful.

Investigator 5: The problem-solving nature of it was just a different level of consideration on what the issues were. […] No question–no question [in response to being asked if they felt the QRI had an impact]. So this language that I'm speaking now is a direct result of that thinking. I don't think we'd delineated it to the same extent then at all. It seems very straight forward and obvious now […]—but this was a completely different world to us. We were just clinicians in a clinic, saying “there's a trial here […]—do you want to go in it?” […] I expected, in the beginning, I'll be honest—I expected “We'll do all this talk and it won't make a difference”—but it did make a difference.

One informant stood apart, having indicated that the feedback of QRI findings had not been “surprising,” although still mentioned specific issues that had compromised recruitment in sites other than their own:

Investigator 9: There's nothing that came as a surprise, but I think it was good. I mean, I think it had an impact. I think recruitment suddenly improved somewhere else.

Interviewer: In what way do you think?

Investigator 9: Well, I think people realized that they were overselling [treatment x] and didn't realize. [Later] But if we get [another study], I think it's very important to have it [QRI] at centers.

Several informants highlighted the necessity for chief investigators, CTUs, and recruiters to engage with the QRI and for its processes to be integrated with the trial to facilitate success:

Investigator 5: The key thing for me was getting the chief investigator and the team around them engaged with it. It will never work if the chief investigator doesn't get it.

Some questioned whether QRI actions had reached all sites, particularly those less engaged with the RCT, but there were also suggestions that feedback/training was unlikely to be restricted to sites the QRI team directly worked with (Web Appendix 2). When asked to summarize their impressions of what the QRI entailed, most emphasized the feedback/training elements, informed by audio-recordings of recruiters' actual practices. Informants tended to discuss the QRI as a “complex intervention,” hesitating to pin-point any one aspect that was more effective than another:

Investigator 5: The whole is greater than the sum of its parts.

Investigator 2: I wouldn't want to put it down to one particular thing. I think it was a combination of things that had an effect. […] I think it's much better if it's integrated into the whole structure.

## 4 Discussion

This article reports the first empirical evaluation of the QRI—an intervention designed to optimize recruitment in challenging RCTs. The QRI's impact on recruitment was examined in five RCTs from oncology and surgical specialties. The QRI identified issues that appeared to undermine recruitment to most of the RCTs, particularly in relation to how the RCT was communicated to patients. QuinteT Recruitment Intervention actions sought to address these difficulties through feedback/training, dissemination of written guidance, and changes to patient-facing documentation. There was evidence of improvement in randomization success after intervention in three RCTs; the two RCTs that did not see an improvement both recruited above target throughout their durations (and had received up-front/prior QRI recruitment training). The QRI also identified issues around approaching eligible patients in some RCTs, resulting in QRI actions designed to improve opportunities for approaching patients. The numbers of eligible patients approached per site/month significantly increased in two trials that specifically implemented these QRI actions. The number of patients approached did not change, or significantly decreased, in the postintervention periods of other RCTs without these QRI actions. Trialists perceived a change in recruitment practices after QRI actions, particularly in relation to how they communicated with patients, with most reporting that the QRI had unearthed issues they had not previously considered. Full integration of the QRI into the RCTs, with support from chief investigators and CTUs, was deemed integral to the QRI's success.

The study had several limitations. We cannot establish cause-effect relationships between QRI actions and recruitment and cannot account for other factors that might have explained changes observed. To minimize these limitations, we examined preintervention/postintervention periods at the level of each site within each trial, rather than defining blanket preintervention/postintervention cutoff periods at the overall trial level. The improvements in randomization success in multiple RCTs support the notion that the QRI actions were genuinely influencing and changing recruitment, although it is possible that recruiters naturally improved their practices over time. The changes observed are unlikely to be due to spontaneous improvements, given that the QRI identified issues that recruiters were not aware of. Furthermore, QRI actions (and improvements) occurred more than 1 year into the recruitment periods of some RCTs, by which point recruiters' practices had likely stabilized.

The evaluation was only undertaken with five UK RCTs and thus would be strengthened by inclusion of more trials spanning a diversity of settings. There were also limitations in the quality and completeness of data available, leading to several a priori assumptions. We assumed that QRI actions affected all recruiters in any given site, and that all sites and recruiters had been exposed to every “study-wide” QRI action (e.g., “tips and guidance” documents). The large RCTs had many centers that never directly engaged with the QRI, but we counted their recruitment activity as “postintervention” following any study-wide QRI actions. These limitations are likely to have resulted in a more conservative estimate of QRI impact, as recruiters not truly exposed to QRI actions may have diluted the “postintervention” effect. The qualitative evaluation was limited by the possibility that informants' responses were influenced by knowledge that the interviewer was affiliated with QRI researchers who worked on their trials. As such, the interviews primarily focused on examining how the QRI operated in each RCT, rather than trialists' perceived value of the QRI.

Strengths of the study included the use of mixed methods, which allowed for a detailed understanding of whether (and how) the QRI was likely to have impacted recruitment. The independent documentary analysis and qualitative interviews triangulated our understanding of the potential mechanisms of change in each RCT, and thus plausibility of effect.

The QRI has previously been recognized as a promising intervention, in a field that is largely devoid of effective solutions to addressing recruitment issues [28], [29], [30]. When faced with the pressures of improving recruitment, trialists often try multiple strategies concurrently, making evaluation difficult. Randomized evaluations have tended to test relatively simple interventions, such as altering patient information materials or arranging site visits [31], [32], [33], [34], [35], [36]. These have largely been shown to have little impact on recruitment, although increasing patients' knowledge about the clinical condition, telephone reminders, monetary incentives, and “opt out” processes can be effective [7], [8]. However, most of these strategies are unlikely to improve recruitment in complex trials involving life-changing conditions or very different interventions, as considered in this evaluation.

The extensive literature on reasons for poor recruitment supports plausible mechanisms of action for the QRI. Recruiters can experience complex intellectual challenges, relating to struggles with equipoise, role conflict, and concerns about their relationships with patients [37], [38], [39]. Retrospective analyses of reasons for trial failure have also indicated that most issues are “preventable,” with recruiters' and patients' preconceptions about treatments frequently underpinning reasons for discontinuation [40]. The QRI recognizes that recruiters' attitudes, beliefs, and skills can undermine recruitment at several stages from identifying eligible patients through to approaching them and presenting the option of trial participation. Although not exclusively a “recruiter-targeted” intervention, most QRI actions are geared toward altering recruiters' practices through guidance and feedback. Other “recruiter-targeted” interventions have taken the form of one-off training [41], although those that have shown positive effects have been context specific [42], [43]. Similar to the QRI, one potentially transferable initiative trained recruiters to elicit and respond to patients' views about trial participation; this appeared to be associated with recruitment success when evaluated in a single trial [44]. The theoretical and empirical literature on clinicians' learning underlines the importance of specific, preplanned, data-driven feedback [45], [46]. In particular, cognitive learning theory proposes that individuals learn by organizing and relating new information to existing knowledge, with feedback presented as one way of achieving this [47].

Application of the QRI to a growing number of RCTs has provided opportunities to identify common recruitment issues [37], [38], [48], [49] and design pre-emptive training [26]. Randomized controlled trials that have most recently integrated the QRI have included up-front training/guidance, as in RCT 5, which did not see a significant improvement in randomization success, although the numbers of patients approached per site/month increased following QRI actions designed to address issues around approaching patients. It is possible that the up-front training may have enhanced recruitment in the “preintervention” period, or RCT 5 might have recruited well without the QRI. Randomized controlled trial 2, which had prior exposure to QRI actions, also recruited above target with no notable issues. Further work is required to investigate the role of up-front generic training versus responsive feedback and any sustainable effects of QRI feedback. Prerecruitment training might address some generic issues (e.g., suggestions for explaining randomization) but is unlikely to address more complex challenges such as equipoise communication, which can be disrupted by trial-specific contextual factors.

Future evaluations of the QRI need to include individual recruiter-level data. Examination of the cost-effectiveness of the QRI would also be valuable, given that the QRI requires time and resources (although likely to be small relative to the costs associated with suboptimal recruitment) [13] and should consider the transferability of QRI feedback/training to other RCTs. Ideally, future evaluations of the QRI should incorporate randomization. Randomizing sites in a given RCT to receive the QRI (or no QRI) would be challenging due to the “ripple effect” of QRI actions and cross-site contamination. Randomization could occur at the RCT level, although this would be practically difficult and require large numbers of trials. Despite these complications, the promising evidence needs to be substantiated by future more robust evaluations of the QRI and its components.

## 5 Conclusion

The QRI supported recruitment in five challenging RCTs, all of which successfully achieved their recruitment targets. There was strong evidence that QRI actions improved randomization success in the RCTs, with the exception of two RCTs that had received prerecruitment QRI training/support, which recruited well from the outset. Improvement in the number of eligible patients approached was also apparent in two RCTs where these issues were specifically addressed. Tailored feedback to recruiters was at the heart of the QRI actions and was perceived by recruiters to have had an impact on their practices. While more robust evaluation is required, there is evidence that the QRI has a positive effect on recruitment in a range of RCTs.

## Supplementary data

Data Profile
