# Supplementary material for: The QuinteT Recruitment Intervention supported five randomized trials to recruit to target: a mixed-methods evaluation
Source: J Clin Epidemiol. 2019 Feb;106:108–20. doi: 10.1016/j.jclinepi.2018.10.004 (PMC6355457; doi:10.1016/j.jclinepi.2018.10.004)
Supplement: Supplementary Information [file mmc2.docx]

**Supplementary information 1**

All RCTs that had collaborated with Quintet between 2005-2017 were assessed for eligibility, for potential inclusion in the evaluation. Not all RCTs that had collaborated with Quintet had implemented the QRI, as the intervention had not stabilised until 2012 (the year it was first named). We nonetheless still assessed all RCTs collaborations, as there was a possibility that the core QRI components had been implemented pre-2012 (even if this was not described as a ‘QRI’).

**Supplementary Information 2**

Information recorded for each RCT included: its design; stage (internal/external pilot or main); comparison groups; recruitment targets; recruitment outcomes (final figures, date recruitment closed, or the date the internal/external pilot ended), and any changes to trial design/targets. Information collected about the QRI integrated in each RCT included: the start and end date of QRI Phase 1/Phase2; key issues found to compromise recruitment (Phase 1 findings), and the timing and nature of QRI-actions to optimise recruitment (Phase 2).

**Supplementary Information 3**

**QRI follow up interview topic guide**

*The purpose of this interview is for us to find out about how the QRI went. We’re interested in what worked and what didn’t work.*

1. **Background**

- What was your role in [trial]?
- Can you tell me a little bit about how the trial came about, its aims, and rationale

1. **Details of RCT**

- Was recruitment anticipated to be problematic? If yes, why?
  - (If yes) had you planned any strategies/methods to mitigate recruitment difficulties?

1. **Collaboration with QuinteT**

- How did the collaboration with QuinteT come about?
- How easy was it to integrate the QRI into the trial?
  - Could anything have been done to help make this process easier?

1. **About trial recruitment**

- How did recruitment go?
- What were the main hurdles to recruitment to [trial]?
- Did you have any plans to mitigate these (other than implementing the QRI)?
- Were you able to overcome these or mitigate their effect? If so, how?

*Probes – more centres? Changes to study design? Other things?*

1. **About the QRI**

- If you were to explain the QRI to a colleague, what would you say it was?
- Thinking about the QRI, what was done in [trial] (and when?)?
  - Probe: Did you receive summaries of the emerging QRI findings?
  - Probe: What was done in relation to identified difficulties?
    - Group feedback session?
    - Individual written feedback to recruiters?
    - Recruitment tips?

1. **What worked…what didn’t work**

- What kind of impact, if any, do you think the QRI interventions had on recruitment?
- How do you think the QRI achieved this impact?
- Were there specific aspects of the QRI that were particularly helpful? [Explore]
- Were there aspects of the QRI that were less helpful? [Explore]
- Can you think of anything else, in terms of support, that might have been helpful?
- Were any other strategies implemented? [If yes] How did these come about? (Who recommended? What prompted?
- Thinking about your practice …did the QRI make a difference to your practice specifically?
  - If not, why do you think this was?
  - If so, how do you think the QRI brought about this change? (Probe: attribute to any particular QRI activity? E.g. feedback? Tip documents? Something else?)
  1. **Have you had any feedback from colleagues regarding the QRI?**

If yes, what? *Explore*

1. **Conclusions and thinking ahead**

- What were your expectations for the QRI? Were these met?
- If we were to re-do the QRI in [trial], do you have any thoughts on what we might do differently?
- Do you see a place for a QRI in future trials that you might lead? And/or in other areas?

**Supplementary information 4**

**Application of exclusion criteria to reach final sample of RCTs included in the evaluation**


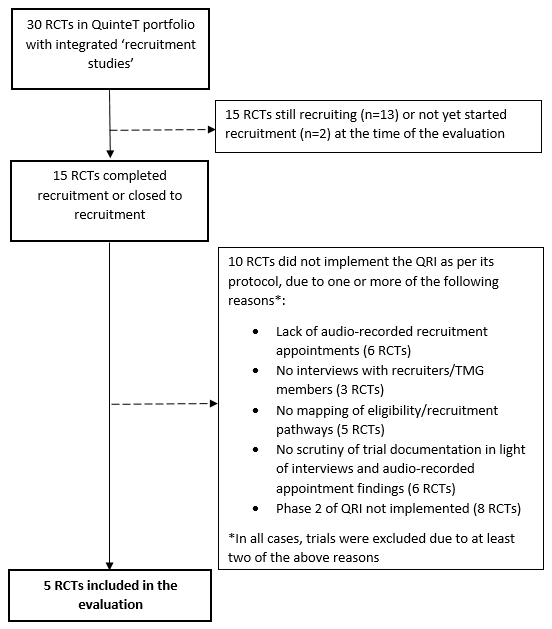


**Web appendix 1**

*Investigator 1: We’ve learnt a huge amount about recruitment along the way, and how to do it, and what equipoise means. So we’ve learnt all from [the QRI] - how to do all that.….and, it’s been a very interesting clinical learning experience...*

*Investigator 2: [Having discussed opening new RCT sites]…An attempt to improve recruitment in our existing centres (was to) adopt new methods, as a consequence of advice from QuinteT. And I would say, unequivocally, the most successful of those two strategies was to improve recruitment in our existing centres, rather than to expand the number of centres..*

**Web appendix 2**

*Investigator 4: [On discussing whether the QRI improved recruitment] It is extremely difficult to know for certain, but I think it did. It did for me, for sure. We had a limited number of sites, so there were individual sites that had their individual obstacles. There were some sites that were very hard to reach, and I suspect the QRI got nowhere near those people. I think that’s an issue with the team.*

*Investigator 2: I think it would be incorrect to assume that the influence of the QRI is limited to those centres you visited. […] What I was doing was using my experiences at all possible occasions to influence and help my colleagues who are in other recruiting centres by talking to them, by meeting with them, by going to visit them, so the influence of the QRI process is not confined to centres you visited.*
